# Is there a role for thoracic aortic calcium to fine-tune cardiovascular risk prediction?

**DOI:** 10.1007/s10554-012-0055-z

**Published:** 2012-04-24

**Authors:** Marc Hartmann, Clemens von Birgelen

**Affiliations:** 1 Department of Cardiology, Thoraxcentrum Twente, Medisch Spectrum Twente, Haaksbergerstraat 55, 7513 ER Enschede, The Netherlands; 2 MIRA Institute, University of Twente, Enschede, The Netherlands

**Keywords:** Thoracic aortic calcification, Coronary artery calcium, Computed tomography, Risk prediction, Calcium score

## Abstract

Screening asymptomatic subjects to streamline measures for the prevention of cardiovascular events remains a major challenge. The established primary prevention risk-scoring methods use equations derived from large prospective cohort studies, but further fine-tuning of cardiovascular risk assessment remains important as 25 % of individuals with low estimated risk may experience cardiac events. Independent studies provided evidence that extended risk assessment using coronary artery calcium quantification may improve risk stratification as it can lead to reclassification of persons at increased risk. Particularly in intermediate-risk subjects, coronary artery calcium scoring can help to correctly identify individuals at highest risk. Data on the extent of calcification of the ascending and descending thoracic aorta might be useful for additional cardiovascular risk stratification. Future analyses and studies will be required to answer the question of whether the implementation of such data may allow further fine-tuning of cardiovascular risk prediction in specific subpopulations—for instance in women or men with an increased risk of stroke and/or symptomatic peripheral vascular disease.

Cardiovascular events are important causes of death and disability. Primary prevention to lower the incidence of such events is of paramount importance. Nevertheless, screening asymptomatic subjects to streamline preventive measures remains a major challenge [1]. Classical cardiovascular risk factors such as age, gender, cholesterol levels, blood pressure, and smoking are traditionally employed to assess the individual risk status, to trigger lifestyle modification, and to guide drug prescription in order to prevent cardiovascular events. Ideally, a predictor of the overall risk of cardiovascular events should be based on a robust multifactorial model [2–5]. The established primary prevention risk-scoring methods use equations derived from large *prospective cohort studies*, such as the European Systematic Coronary Risk Evaluation Project (SCORE) [2], the German Prospective Cardiovascular Münster (PROCAM) study [3], and the US-American Framingham Heart and Offspring Studies [4, 5]. These estimated risk values even appear to be related to coronary plaque progression rates [7]. Risk prediction models may be most favorable for the continent, subcontinent, or even country, from which an underlying data set is derived, which is the reason for the current coexistence of several cardiovascular risk scores [2–5]. Current guidelines on dislipidemia use the estimated individual risk from such scoring systems to guide lipid-lowering therapy with statins in primary cardiovascular prevention [6]. The abovementioned facts underline the importance of *prospective cohort studies* to gain information about population-based cardiovascular event risks, which is required to develop evidence-based clinical guidelines that have major impact on our daily clinical practice.

*Fine*-*tuning* *of cardiovascular risk* assessment remains important as 25 % of individuals with low estimated risk may experience cardiac events [1]. Independent studies provided evidence that extended risk assessment using coronary artery calcium (CAC) quantification may improve risk stratification as it can lead to reclassification of persons at increased risk [8–12]. One example of such large, population-based cohort studies is the Heinz Nixdorf Recall Study (4814 participants from the Ruhr metropolitan area in Germany), which investigates the ability of subclinical CAC quantification with electron-beam computed tomography scanning to predict the risk of major cardiovascular events at five-year follow-up above and beyond traditional cardiovascular risk factors [1, 11, 13]. This study demonstrated that a higher cardiovascular risk burden was associated with higher CAC scores and that CAC scoring improved risk stratification, discrimination, and reclassification [1, 11]. Particularly in intermediate-risk subjects, CAC scoring can help to correctly identify individuals at highest risk, which might contribute to reducing the number of coronary events in the general population [1, 11]. A representative US-American population, the Multi-Ethnic Study of Atherosclerosis (MESNA), also demonstrated an incremental prognostic value of absolute CAC scores over several traditional cardiovascular risk factors [14].

In the current issue of the journal, Raimund Erbel and his coworkers present interesting thoracic aortic calcification (TAC) data derived from the Heinz Nixdorf Recall cohort study [15]. The extent of calcification of the ascending and descending thoracic aorta, as determined with electron-beam computed tomography, was significantly associated with CAC burden [15]. While TAC largely shared cardiovascular risk factors with coronary artery disease, TAC was independently related to CAC [15].

In fact, these data from a general population in Western Europe [15] are very welcome. Data on TAC in large, general populations are scarce and were previously merely available from US-American populations [16–19]. Wong et al. studied in 2303 asymptomatic participants the ability of CAC and TAC to predict coronary heart disease and cardiovascular events and found that CAC, but not TAC, showed a strong relation with coronary heart disease and cardiovascular events [16]. However, in another US-American study, the MESNA study (n = 6814 of four ethnic groups), TAC was found to be a strong predictor of CAC independent of cardiovascular risk factors [17, 18]. Santos et al. even demonstrated in a cohort of 8401 asymptomatic, predominantly white US-Americans, undergoing cardiac risk factor evaluation and scanning with electron-beam computed tomography, that the presence of TAC was associated with all-cause mortality, a relation that was independent of conventional cardiovascular risk factors and the presence of CAC [19]. In a Dutch high-risk population of 958 heavy smokers, Jacobs et al. found that CAC was a stronger predictor of cardiovascular events than TAC [20]. However, in this quite specific subpopulation of a lung cancer screening trial, TAC was stronger associated with vascular disease and events such as stroke as well as the occurrence of aortic aneurysms and occlusive peripheral arterial disease [20].

The present report from the Heinz Nixdorf Recall Study provides valuable computed age and gender-specific percentile curves for TAC in a Western European general population [15]. These percentiles may help to interpret individual thoracic calcification values and might be useful for additional cardiovascular risk stratification. Future analyses and studies will be required to answer the question of whether the implementation of these data may allow further *fine*-*tuning of cardiovascular risk prediction* in specific subpopulations—for instance in women or men with an increased risk of stroke and/or symptomatic peripheral vascular disease.
